# Recovery and prognostic value of myocardial strain in ST-segment elevation myocardial infarction patients with a concurrent chronic total occlusion

**DOI:** 10.1007/s00330-019-06338-x

**Published:** 2019-07-26

**Authors:** Joëlle Elias, Ivo M. van Dongen, Loes P. Hoebers, Dagmar M. Ouweneel, Bimmer E. P. M. Claessen, Truls Råmunddal, Peep Laanmets, Erlend Eriksen, Jan J. Piek, René J. van der Schaaf, Dan Ioanes, Robin Nijveldt, Jan G. Tijssen, José P. S. Henriques, Alexander Hirsch, on behalf of the EXPLORE investigators

**Affiliations:** 1 Amsterdam UMC, Heart Center, Department of Cardiology, Amsterdam Cardiovascular Sciences, University of Amsterdam, Meibergdreef 9, 1105 AZ Amsterdam, The Netherlands; 2 Sahlgrenska University Hospital, Gothenburg, Sweden; 3 North Estonia Medical Center, Tallinn, Estonia; 4 Haukeland University Hospital, Bergen, Norway; 5 Onze Lieve Vrouwe Gasthuis, Amsterdam, The Netherlands; 6 University Medical Center St Radboud, Nijmegen, The Netherlands; 7 Erasmus Medical Center, Rotterdam, The Netherlands

**Keywords:** Coronary occlusion, Percutaneous coronary intervention, ST elevation myocardial infarction, Magnetic resonance imaging

## Abstract

**Objectives:**

Global left ventricular (LV) function is routinely used to assess cardiac function; however, myocardial strain is able to identify more subtle dysfunction. We aimed to determine the recovery and prognostic value of featuring tracking (FT) cardiovascular magnetic resonance (CMR) strain in ST-segment elevation myocardial infarction (STEMI) patients with a concurrent chronic total occlusion (CTO).

**Methods:**

In the randomized EXPLORE trial, there was no significant difference in global LV function after percutaneous coronary intervention (PCI) of the CTO, compared with no-CTO PCI, post-STEMI. In the current study, we included 200 of the 302 EXPLORE patients with a baseline CMR, of which 180 also had 4-month follow-up (serial) CMR. Global longitudinal strain (GLS) was calculated from 3 long-axis views. Global circumferential strain (GCS) and segmental strain were calculated from 3 short-axis views (basal, mid, and apical).

**Results:**

Global strain significantly improved at 4 months (GLS ∆ − 1.8 ± 4.3%, *p* < 0.001; GCS ∆ − 1.7 ± 4.7%, *p* < 0.001); however, there was no treatment effect of CTO-PCI on strain recovery. GLS was a significant predictor for 4 months of LV ejection fraction (*p* = 0.006), incremental to other CMR parameters including infarct size. For mortality, infarct size remained the strongest predictor. On regional level, segmental strain independently predicted recovery in the dysfunctional segments (*p* < 0.001).

**Conclusions:**

Global and segmental myocardial strains significantly improved over time, with no effect of CTO-PCI. Global strain was associated with outcome and segmental strain was an independent predictor for regional LV recovery in the dysfunctional CTO territory. Further research is needed to determine the additional prognostic value of strain beyond routine CMR parameters.

**Key Points:**

*• In STEMI patients with a concurrent CTO, strain significantly improves over time, regardless of CTO-PCI.*

*• Global strain is an independent predictor for functional recovery, incremental to infarct size, LVEF, and clinical parameters.*

*• Segmental strain was able to predict the recovery of wall thickening, incremental to transmural extent of infarction.*

**Electronic supplementary material:**

The online version of this article (10.1007/s00330-019-06338-x) contains supplementary material, which is available to authorized users.

## Introduction

Cardiovascular magnetic resonance (CMR) is frequently used for non-invasive assessment of global left ventricular (LV) function and infarct size, which can be used as surrogate endpoints to predict clinical outcome of ST-segment elevation myocardial infarction (STEMI) patients [1]. However, more subtle but important contractile changes might not be detected, as these do not always lead to a decline in global LV function. Currently, new techniques, such as myocardial strain parameters, are therefore gaining more interest as they are able to identify subtle myocardial deformation, are less subjective, and are less experience-dependent [2]. The reference method for the quantification of cardiac LV deformation is myocardial tissue tagging; however, this technique requires additional image acquisition and post-processing analysis and is therefore more time-consuming [3]. CMR-based feature tracking (FT) is novel and highly correlated with myocardial tissue tagging, but it is more clinically feasible as it uses the steady-state free-precision cine images acquired with standard CMR protocols [2, 4]. FT-CMR provides a fast and accurate assessment of myocardial strain by following the border tracking over time and defining the relative change in length of the myocardial segment [5]. FT-CMR is reproducible and comparable values are provided with different software methods [6, 7]. Global longitudinal strain (GLS) and global circumferential strain (GCS) are suggested to be more sensitive in detecting myocardial contractility changes before there is a change in global LV parameters and also to be less variable when compared with LV ejection fraction (LVEF) and wall motion analysis [8].

In STEMI patients with a concurrent chronic total occlusion (CTO), neither the recovery and prediction of outcome using global and segmental strains have thus far been examined nor the effect of additional percutaneous coronary intervention (PCI) of the CTO on the recovery of strain. Currently, the treatment of the (accidently) found concurrent CTO in STEMI patients during primary PCI remains controversial. Observational data have suggested beneficial effects of CTO-PCI [9]. However, the first randomized Evaluating Xience and left ventricular function in PCI on occlusiOns afteR STEMI (EXPLORE) trial, which included STEMI patients, after successful primary PCI, with a CTO and randomized patients to either CTO-PCI or no-CTO PCI, showed no beneficial effect on global LV function (LVEF and LVEDV) [10]. We used the patients included in the EXPLORE trial to (1) investigate the recovery in global and segmental strain parameters from baseline to follow-up, (2) study the effect of CTO-PCI on this recovery, and (3) determine the incremental prognostic value of global and segmental strains in predicting functional and clinical outcomes.

## Methods

In the current sub-study from the EXPLORE trial, we included all patients who underwent a baseline CMR (200 of the 302 patients), of which 180 patients also had a 4-month CMR (serial CMR). This cohort of patients with serial CMR has been described before [11]. In these 200 patients, offline strain analysis was performed; the other patients (*n* = 102) were excluded because they lacked a baseline CMR (a baseline CMR was not mandatory in the study protocol). Details regarding the design and results of the EXPLORE trial were previously reported [10, 12]. In short, the randomized multi-center clinical EXPLORE trial included 302 STEMI patients with a concurrent CTO and randomized them in a 1:1 ratio to CTO-PCI within 7 days after primary PCI (*n* = 148) or to a conservative strategy (no-CTO PCI) for at least 4 months (*n* = 154). Important inclusion criteria were the following: a concurrent CTO in a non-infarct-related artery found during successful primary PCI for STEMI and the CTO had to be located in a coronary vessel with a reference diameter of at least 2.5 mm. Among the exclusion criteria were the following: > 48 hemodynamic instability and factors precluding reliable CMR imaging (atrial fibrillation, severe renal insufficiency, and pacemakers or implantable cardioverter-defibrillators). Full inclusion and exclusion criteria are summarized in the Supplemental File. CTO definition was a 100% luminal narrowing without antegrade flow. An independent angiography corelab assessed all coronary angiographies. There were no significant differences in the primary outcomes of LVEF and LV end-diastolic volume (LVEDV) at 4 months of follow-up in patients randomized to CTO-PCI compared with no-CTO PCI, nor on LV systolic volume, LV mass, and infarct size.

### CMR protocol

All CMRs were performed on a 1.5-Tesla scanner using a dedicated phased array cardiac receiver coil. ECG-gated steady-state free-precession cine images for LV function imaging were obtained, during repeated breath holds, in long-axis orientation (2-, 3-, and 4-chamber views) and in short-axis orientation covering the left ventricle from base to apex. Late gadolinium-enhanced (LGE) images were acquired using an inversion recovery gradient-echo pulse sequence with slice locations identical to the cine images to identify the size and extent of infarction. Images were acquired at least 10 min after administration of a gadolinium-based contrast agent in a dosage of 0.2 mmol/kg of body weight. Transmurality of scar tissue of the myocardium was assessed in patients who underwent baseline CMR of sufficient quality. To assess viability, transmural extent of infarction (TEI) was used, TEI of 0–50% per segment was considered viable [13].

### Wall thickening

The analysis of segmental wall thickening has been described before [11]. An independent core laboratory, blinded for randomization outcome, analyzed all CMR images (ClinFact Corelab using QMass MR analytical software version 7.6, Medis BV). A 16-segment model, excluding the apex, was used to analyze wall thickening. Endo- and epicardial borders on the end-diastolic and end-systolic images were manually outlined on all short-axis cine slices. Segmental wall thickening was defined as a percentage increase of LV wall thickness during systole compared with diastole. Myocardial segments were considered dysfunctional if wall thickening was less than 45% [14]. Individual segments for each patient were assigned to one of the major coronary arteries using the American Heart Association standardized myocardial segmentation and nomenclature statement [15]. Myocardial segments were assigned to the CTO, infarct, or remote territory using this standard model (in relation to the coronary anatomy scored by the angiographic corelab).

### Strain analysis

Strain measurements were performed offline using the FT-CMR software method of Medis QStrain Software (Medis Medical Imaging Systems, version 2.0.12.2.) (example of the analysis is in the Supplementary File). All three longitudinal-axis views (2-, 3-, and 4-chamber) were used to determine peak GLS. Endocardial contours were manually drawn during end-diastole and end-systole with subsequent automatic tracking during the cardiac cycle. As an example, a cine CMR movie file of the endocardial tracking is available as additional files 1 and 2. For the assessment of GCS and segmental circumferential strain, the corelab contours for the short-axis images were used. Peak GCS was calculated from 3 short-axis views (basal, mid, and apical). For peak segmental strain, short-axis images were used to define the segments according to the 16-segment model after manual insertion of a reference point (delineated at the anterior insertion of the right ventricle). All studies were loaded into the software and analyzed in a random order by one investigator blinded for randomization outcome under supervision of a CMR cardiologist with > 15-year experience (JE, supervisor: AH). The reproducibility of GLS measurements was assessed in 30 CMR scans (15 patients with baseline and follow-up CMR). The intraclass correlation coefficient for interobserver agreement was 0.97 (95% CI 0.89 to 0.99; *p* < 0.001).

### Clinical outcomes

At 4 months and 1, 2, 3, 4, and 5 years, clinical follow-up was collected to assess survival status. Survival data were censored at 5 years or known date of last contact [16].

### Statistical analysis

Data are presented as mean ± standard deviation for continuous variables. Discrete variables are presented as frequencies and percentages. Baseline characteristics were compared using the independent-samples *t* test, or Fisher’s exact probability test in case of binary endpoints. Analyses were performed on intention-to-treat analysis. Changes in GLS and GCS within each group were tested with paired Student’s *t* test. Pearson’s correlation coefficient analysis was used to assess the relationship between the global strain and LV function parameters. Multivariable linear regression was used for testing the contribution of baseline, angiographic, and CMR characteristics in relation to LVEF at follow-up. Stepwise forward selection of variables was used; all variables with a *p* value < 0.05 were included and variables with a *p* value > 0.10 were removed from the model. Cumulative event rates of long-term mortality were estimated using Kaplan-Meier curves, and the Log rank statistic was used for comparing the survival curves. Hazard ratios for long-term mortality were calculated, after verification of the proportional hazard assumption, using Cox proportional hazard regression analyses. We evaluated the recovery of segmental circumferential strain in dysfunctional segments at baseline. Because within 1 patient the regional strain in the different segments is strongly related and not an independent outcome, multilevel analysis was used (linear regression) [17]. The following fixed effects were included: randomization outcome and baseline segmental strain. Multilevel analysis was also used to look for predictors of regional wall thickening recovery, and the following fixed effects were included: baseline SWT, baseline transmural extent of infarction, presence of microvascular obstruction, randomization outcome, and baseline segmental strain. All tests were two-sided, and a *p* value < 0.05 was considered to indicate statistical significance. Statistical analysis was performed with the Statistical Package for Social Sciences software (SPSS version 23.0 for Windows).

## Results

### Baseline characteristics

**Fig. 1:**
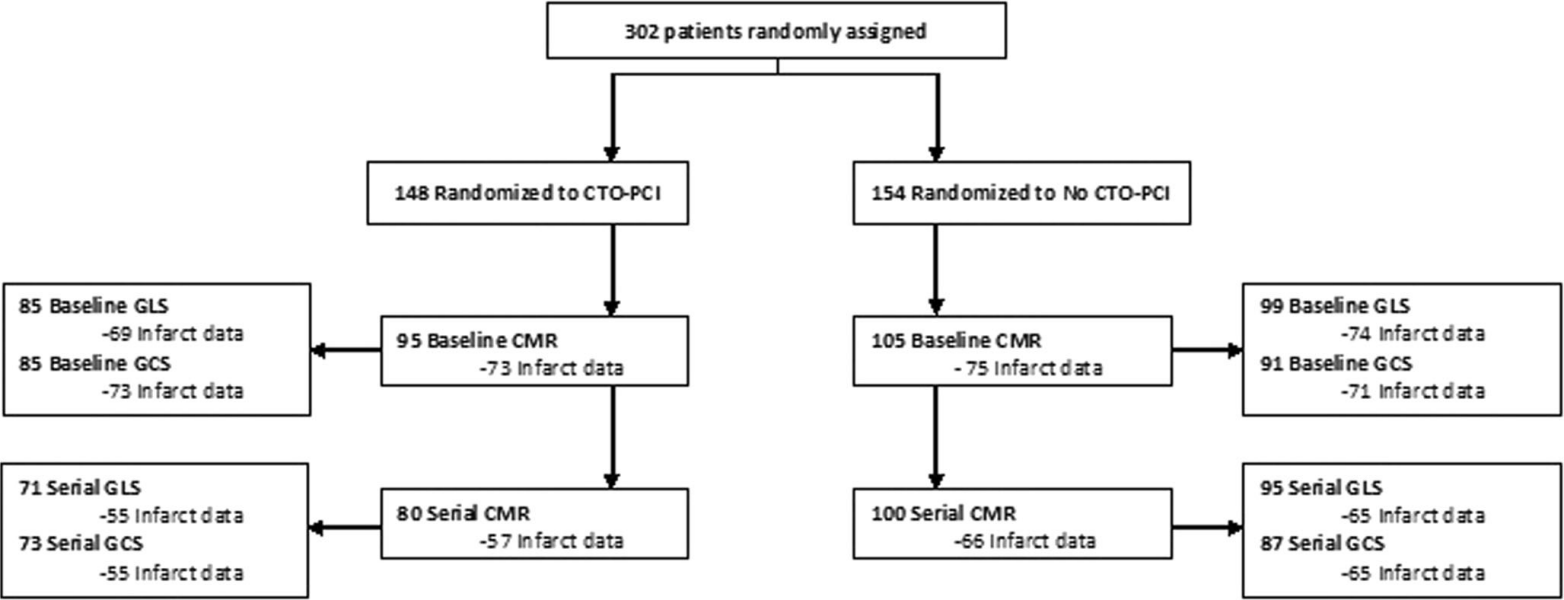
Flowchart of the EXPLORE trial and available cardiovascular magnetic resonance data. CTO = chronic total occlusion; PCI = percutaneous coronary intervention; CMR = cardiac magnetic resonance; GLS = global longitudinal strain; GCS = global circumferential strain

The baseline characteristics of the 200 patients included in this study are shown in Supplement Table 1. Patients had a mean age of 60 ± 10 years and 88% was male. Mean baseline LVEF was 41 ± 12%, LVEDV 103 ± 25 ml/m^2^, and infarct size 12 ± 11 g. In the patients randomized to CTO-PCI, the PCI was performed on day 5 ± 2 after primary PCI. Baseline CMR was performed 4 ± 2 days after primary PCI. Baseline GLS was available in 184 patients and GCS in 176 patients. Serial GLS measurements were available in 166 and serial GCS in 160 patients (Fig. 1). GLS and GCS by FT-CMR were significantly correlated with LVEF, LVEDV, and infarct size (*p* < 0.0001 for all). Changes in strain parameters were also related to change in LVEF; however, change in LVEDV and infarct size showed poor correlation with change in global strain (Supplement Table 2).

### Global myocardial strain at follow-up

**Fig. 2:**
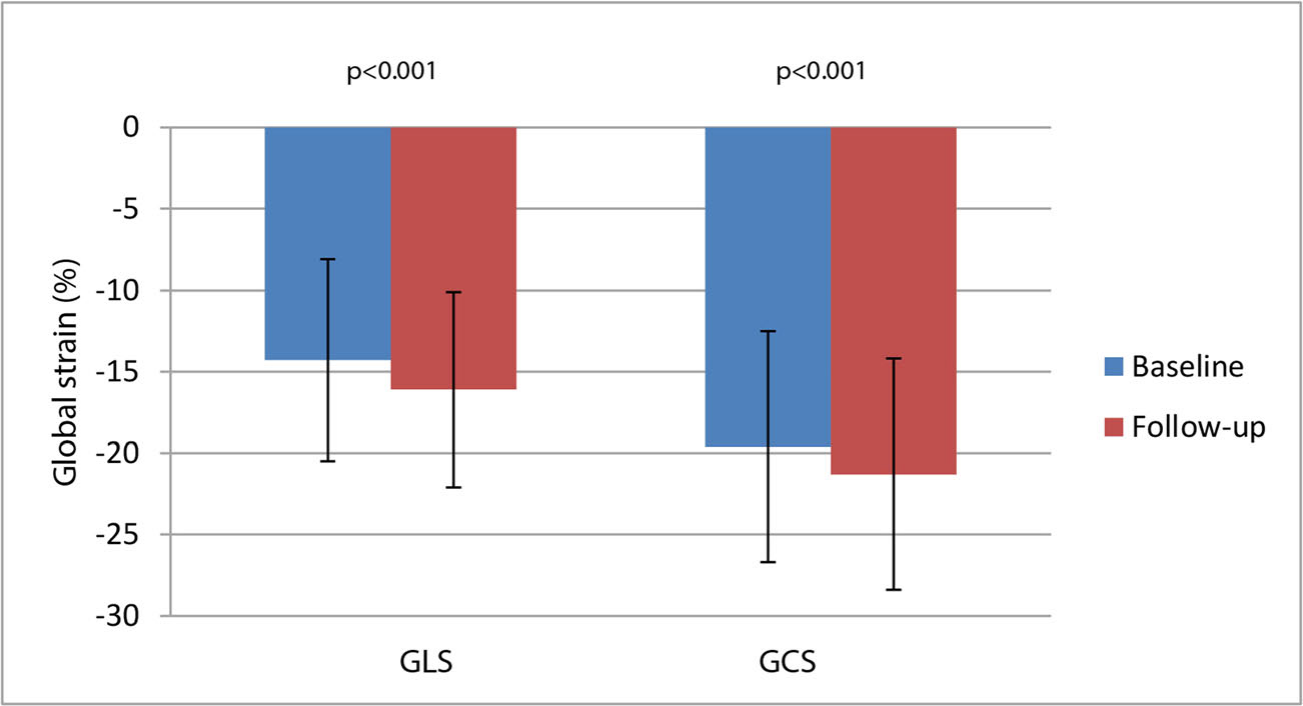
Recovery of global strain in the total cardiovascular magnetic resonance population from baseline to follow-up. Global longitudinal strain (GLS) (left) and global circumferential strain (GCS) (right) from baseline to 4-month follow-up. Whiskers indicate standard deviation

GLS and GCS significantly improved from baseline to 4 months of follow-up (ΔGLS − 1.8 ± 4.3%, *p* < 0.001; ΔGCS − 1.7 ± 4.7%, *p* < 0.001) (Fig. 2). However, there was no treatment effect of CTO-PCI on the recovery of global strain parameters (∆GLS − 2.4 ± 4.2% versus − 1.4 ± 4.3%, *p* = 0.14; ∆GCS − 1.4 ± 4.5% versus − 1.9 ± 4.9%, *p* = 0.55) (Table 1).

**Table 1:** Recovery of global strain comparing CTO-PCI versus no-CTO PCI

GLS (%)	Total (*n* = 166)	*p* value	CTO-PCI (*n* = 71)	No-CTO PCI (*n* = 95)	*p* value*
Baseline	− 14.3 (6.2)		− 13.9 (6.5)	− 14.6 (6.0)	0.53
Follow-up	− 16.1 (6.0)		− 16.3 (5.8)	− 15.9 (6.1)	0.68
Change	− 1.8 (4.3)	< 0.001^†^	− 2.4 (4.2)	− 1.4 (4.3)	0.14
GCS (%)	Total (*n* = 160)	*p* value	CTO-PCI (*n* = 73)	No-CTO PCI (*n* = 87)	*p* value*
Baseline	− 19.6 (7.1)		− 19.5 (7.2)	− 19.7 (7.1)	0.90
FU	− 21.2 (7.1)		− 20.9 (6.7)	− 21.5 (7.4)	0.60
Change	− 1.7 (4.7)	< 0.001^†^	− 1.4 (4.5)	− 1.9 (4.9)	0.55

*Difference between CTO-PCI and no-CTO PCI. Outcomes were analyzed using paired Student’s *t* test. Data are mean ± SD

^†^Significant recovery of strain from baseline to 4-month follow-up

*GLS*, global longitudinal strain; *GCS*, global circumferential strain; *CTO*, chronic total occlusion; *PCI*, percutaneous coronary intervention

### Prognostic value of global strain

**Fig. 3:**
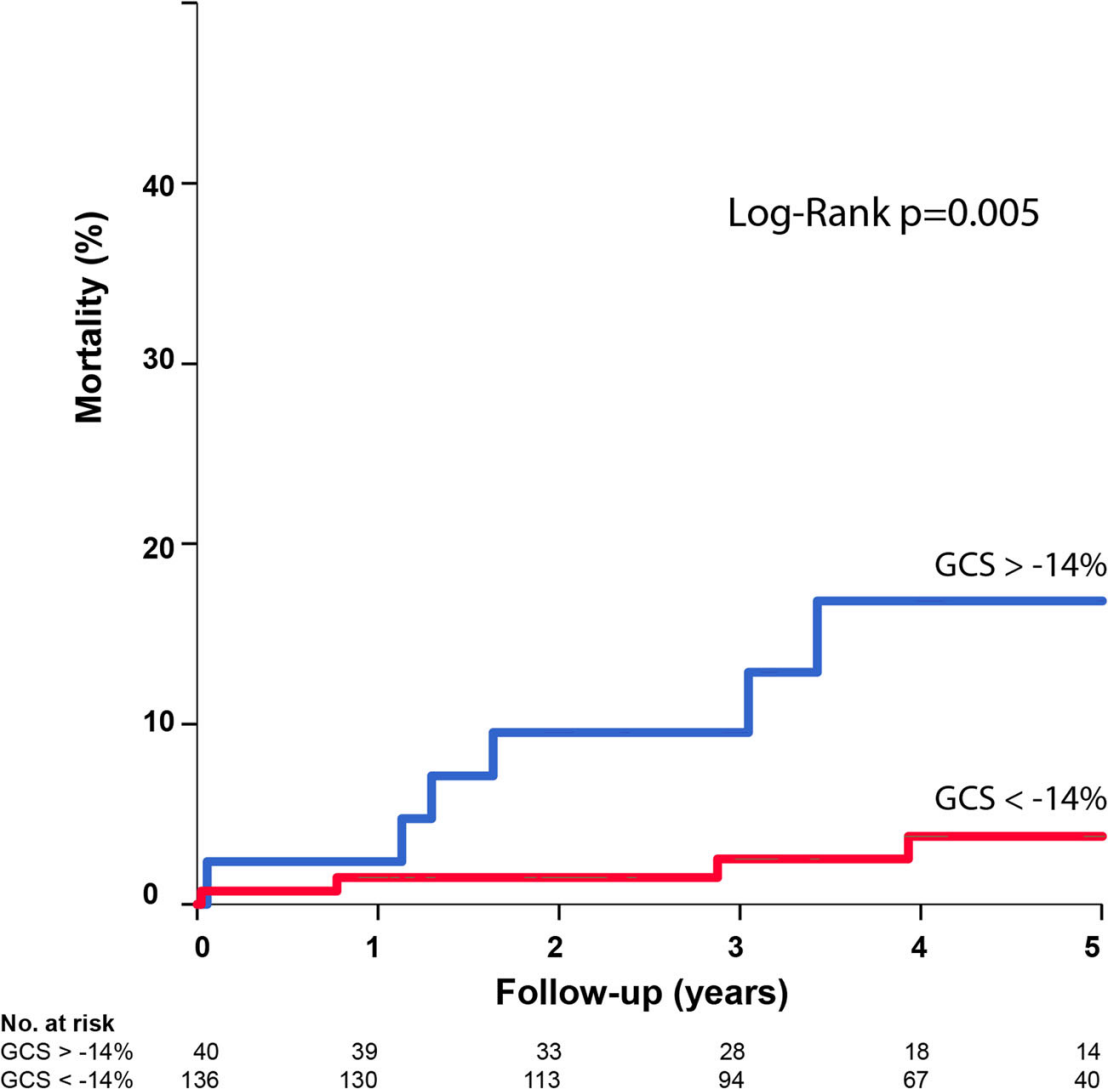
Kaplan-Meier curves representing long-term mortality in patients with GCS < − 14% and patients with GCS > − 14%. Kaplan-Meier estimates of the cumulative event rates

Both GLS and GCS were univariate predictors for LVEF at 4-month follow-up. In multivariate analysis, GLS remained a significant predictor for functional outcome (4-month LVEF) (*ß* − 0.40, 95% CI − 0.68 to − 0.12, *p* = 0.006), together with baseline LVEF, LVEDV, and presence of microvascular obstruction (MVO) (Table 2). By the Kaplan-Meier analysis, the patients in the lowest quartile GCS (GCS > − 14%) had a significantly worse survival compared with patients with GCS < − 14% (Fig. 3). During a median follow-up of 4.0 (2.2–5.0) years, 13 patients died (6.5%). Univariate predictors for long-term mortality were LVEF, LVEDV, and GCS. In stepwise forward multivariate analysis, GCS remained the strongest predictor for mortality (HR 1.17, 95% CI 1.04–1.31, *p* = 0.009) (Supplement Table 3). In 74% of the patients, infarct data was available; when including infarct size and MVO in the model, infarct size was the only significant CMR parameter predicting mortality (HR 1.07, 95% CI 1.03–1.11, *p* < 0.001).

**Table 2:** Prediction of left ventricular ejection fraction at 4-month follow-up (*n* = 129)

	Univariate analysis			Multivariate analysis		
	Beta	95% CI	*p* value	Beta	95% CI	*p* value
Age (years)	− 0.01	− 0.20 to 0.18	0.93			
Male	− 1.31	− 7.29 to 4.66	0.67			
Diabetes	− 8.06	− 13.00 to − 3.12	0.002			
Infarct LAD	− 4.11	− 7.77 to − 0.45	0.03			
CTO LAD	1.84	− 2.50 to 6.18	0.40			
CTO-PCI	0.85	− 2.88 to 4.57	0.65			
Baseline LVEF (%)	0.70	0.60 to 0.81	< 0.001	0.45	0.29 to 0.60	< 0.001
Baseline LVEDV (ml/m^2^)	− 0.22	− 0.29 to − 0.15	< 0.001	− 0.08	− 0.13 to − 0.02	0.005
MVO present	− 6.64	− 10.18 to − 3.10	< 0.001	− 2.48	− 4.81 to − 0.14	0.04
Baseline infarct size (g)	− 0.46	− 0.61 to − 0.30	< 0.001			
Baseline GLS (%)	− 1.20	− 1.41 to − 0.98	< 0.001	− 0.40	− 0.68 to − 0.12	0.006
Baseline GCS (%)	− 1.18	− 1.35 to − 1.00	< 0.001			

Stepwise forward selection of variables was used for multivariable linear regression

*CI*, confidence interval; *CTO*, chronic total occlusion; *LAD*, left anterior descending artery; *LVEDV*, left ventricular end-diastolic volume; *LVEF*, left ventricular ejection fraction; *PCI*, percutaneous coronary intervention; *GLS*, global longitudinal strain; *GCS*, global circumferential strain; *MVO*, microvascular obstruction

### Segmental circumferential strain at follow-up

There were 2560 segments available for serial segmental circumferential strain analysis (from *n* = 160 patients). Segmental strain was significantly recovered from baseline to 4-month follow-up in the dysfunctional segments (wall thickening < 45% at baseline; ∆ segmental strain − 2.4 (8.9), *p* < 0.001; Table 3). However, no significant difference was found between CTO-PCI and no-CTO PCI. Table 3 shows the recovery of segmental strain in the dysfunctional segments in the CTO. In the CTO territory, no significant difference was found on segmental strain between CTO-PCI and no-CTO PCI (∆ segmental strain − 2.5 ± 9.5% versus − 2.5% ± 8.5%, *p* = 0.50).

**Table 3:** Serial cardiovascular magnetic resonance outcomes: recovery of segmental circumferential strain

			CTO territory			Infarct territory		
Dysfunctional segments (SWT < 45%)	Total (*s* = 1504)	*p* value*	CTO-PCI (*s* = 223)	No-CTO PCI (*s* = 275)	*p* value*	CTO-PCI (*s* = 294)	No-CTO PCI (*s* = 316)	*p* value*
Baseline	− 17.2 (9.2)	–	− 17.5 (10.0)	− 16.8 (8.9)	0.41	− 15.8 (8.7)	− 16.3 (9.1)	0.46
Follow-up	− 19.7 (9.4)	–	− 20.2 (9.8)	− 19.3 (9.3)	0.33	− 19.1 (9.3)	− 18.6 (9.7)	0.68
Change	− 2.4 (8.9)	< 0.001^†^	− 2.5 (9.5)	− 2.5 (8.5)	0.50	− 3.2 (9.4)	− 2.3 (9.0)	0.42

*Outcomes were analyzed using multilevel analysis (linear regression); the following fixed effects were included: randomization outcome and baseline segmental strain

^†^Significant recovery of strain from baseline to 4-month follow-up

Data are mean ± SD. *s*, number of segments; *CTO*, chronic total occlusion; *PCI*, percutaneous coronary intervention; *SWT*, segmental wall thickening

### Prediction of wall thickening recovery with segmental strain

At baseline, 59% of the myocardial segments were dysfunctional (wall thickening < 45%). Of the dysfunctional segments, 74% was viable (TEI < 50%) and MVO was present in 13%. In a multivariate analysis, baseline wall thickening, segmental strain, and infarct (TEI) were all statistically significantly related; however, based on the *t* statistic, wall thickening and segmental strain were stronger predictors for wall thickening recovery compared with infarct or MVO. In the CTO territory, 59% of the segments were dysfunctional and 96% of the dysfunctional segments were viable (TEI < 50%). In multivariate analysis, segmental strain was an independent predictor for regional wall thickening recovery, incremental to baseline wall thickening and infarct (coefficient − 0.44, SE 0.14, *t* − 3.10, *p* = 0.002; Table 4).

**Table 4:** Multivariate analysis of predictors of regional recovery (change in wall thickening)

	Coefficient	SE	*t*	*p* value*
Dysfunctional segments (*s* = 1202)				
Segmental strain	− 0.31	0.08	− 3.85	< 0.001
Wall thickening	− 0.55	0.05	− 11.96	< 0.001
Infarct (TEI)	− 0.08	0.04	− 1.74	0.08
MVO present	− 6.43	2.45	− 2.96	0.003
CTO-PCI	4.85	2.45	1.98	0.05
Dysfunctional segments in CTO territory (*s* = 498)				
Segmental strain	− 0.44	0.14	− 3.17	0.002
Wall thickening	− 0.45	0.08	− 5.39	< 0.001
Infarct (TEI)	− 0.12	0.08	− 1.51	0.13
CTO-PCI	7.73	3.06	2.53	0.01

*Outcomes were analyzed using multilevel analysis (linear regression); the following fixed effects were included: baseline segmental wall thickening, baseline transmural extent of infarction, presence of microvascular obstruction, randomization outcome, and baseline segmental strain

*s*, number of segments; *MVO*, microvascular obstruction; *CTO*, chronic total occlusion; *PCI*, percutaneous coronary intervention; *TEI*, transmural extent of infarction

## Discussion

This is the first study evaluating the recovery and value of global and segmental myocardial strains in STEMI patients with a concurrent CTO, and the first to determine the effect of CTO-PCI on strain recovery. The main findings are the following: (1) Global and segmental strains improved over time, with no significant treatment effect of CTO-PCI; (2) GLS was a significant predictor for functional outcome incremental to infarct size, LV function, and clinical parameters; however, for mortality, infarct size remained the strongest predictor; and (3) segmental strain is incremental to TEI in predicting regional wall thickening recovery, especially in the CTO territory.

### Global strain

Although strain significantly recovered at follow-up, we did not find a beneficial effect of CTO-PCI compared with no-CTO PCI on this recovery, which is consistent with the main EXPLORE trial results. FT-CMR strain has substantial clinical potential to be of diagnostic and prognostic value. Global strain measurements are highly reproducible with good to excellent intra- and inter-reproducibility and analysis appears not to be influenced by the level of training [7, 18]. However, only limited data is available on reference values of strain in healthy subjects. LVEF and LVEDV are frequently used in predicting patient prognosis; nonetheless, they are limited as contractility is not measured and they are affected by patient heart rate, loading conditions, and heart valve function [19]. We found that GLS is an independent predictor for global LV function, incremental to LVEF and LVEDV. In a beating heart, there are two longitudinal movements, shortening and lengthening (mainly reflecting GLS), and two transversal movements, narrowing and widening (mainly reflecting GCS). The cardiac cycle consists of different phases as described in more detail by Torrent-Guasp et al [20]. In short, (1) a decrease in the transversal diameter of the base caused by basal loop contraction (narrowing movement), (2) a decrease in the longitudinal axis (shortening movement), (3) an increase in the longitudinal axis (lengthening movement), and (4) an increase in the transversal diameter of the base are conditioned by the relaxation of the ventricular walls and widening movement [20]. GLS is mainly determined by subendocardial myofibers (the basal loop), which are more prone to early myocardial damage before global LV parameters are affected. Therefore, diminished GLS is an early marker of LV dysfunction. This could explain why GLS was the best predictor of LV preservation at follow-up. GCS is largely based on contraction of circumferential myofibers, which stay mostly preserved during early LV deterioration and serve as a restriction to prevent expansion of the LV [21].

For mortality, infarct size remained the strongest predictor. Previous reports regarding the prognostic value of global strain have been conflicting. In a study with 470 ischemic and non-ischemic cardiomyopathy patients, GLS was a strong predictor for mortality incremental to LVEF and infarct size [22]. In 74 STEMI patients, GCS was able to predict preservation of global function (LVEF > 50%) at follow-up similar to infarct size [23]. However, in a study of 65 STEMI patients, GLS was not able to predict adverse LV remodeling [5]. In another study, GLS did not improve risk stratification compared with baseline characteristics and CMR indices in 323 STEMI patients [24]. In the largest strain study thus far (1,235 MI patients), GLS did have incremental prognostic value over LVEF and infarct size to predict mortality [25]. Nonetheless, most studies performed are of small sample sizes and with relative short follow-up.

### Segmental strain

Segmental strain significantly recovered at follow-up. Yet, there was no effect of CTO-PCI, compared with no-CTO PCI, on global nor on segmental strain recovery. However, we have previously shown that wall thickening significantly improves after CTO-PCI compared with no-CTO PCI in the dysfunctional CTO territory [11]. Although strain has the potential to detect more subtle regional differences, we could not reproduce this finding with segmental strain, although the number of segments was relatively low. Furthermore, it is important to mention that previous studies reported high degrees of measurement variability and relatively poor segmental strain reproducibility [26, 27]. Therefore, segmental strain data may be less reliable and its use in clinical practice should be done with caution.

We did find that segmental strain was a strong predictor for wall thickening recovery, incremental to infarct, especially in the dysfunctional CTO territory. This finding is consistent with previous data: in 45 STEMI patients, segmental strain was incremental to infarct and MVO in predicting recovery of wall thickening [28]. However, another study in STEMI patients showed that segmental strain was only a mild predictor of wall thickening recovery and inferior compared with infarct [29]. Furthermore, segmental strain and infarct transmurality are also related, as segmental strain is a predictor for infarcted segments [30]. Therefore, the incremental predictive value and exact relation of segmental strain and infarct needs further examination as, different from infarct (TEI), segmental strain can be measured without the use of a contrast agent, making it a possible alternative in patients with contrast allergy or renal failure.

### Clinical use of myocardial strain assessment

In our high-risk patient population, new risk stratification parameters may be relevant to adequately select patients for CTO revascularization. None of the randomized CTO trails conducted showed a beneficial effect of CTO-PCI on clinical outcome nor on LVF, although ischemia and viability testing prior to inclusion were not mandated. It remains to be determined what the optimal patient selection threshold for CTO revascularization is and whether optimal selection will indeed lead to improved outcomes. Further studies are needed to investigate the true value of (stress) FT-CMR strain in clinical evaluation and whether it can be used as a diagnostic and prognostic tool to select patients that might benefit from PCI.

### Limitations

There are several limitations applicable to this study. Unfortunately, not all CMRs were suitable for strain analysis and not all patients underwent baseline CMR. Furthermore, infarct data was not available in all patients and only 4% of the dysfunctional segments in the CTO territory were non-viable (TEI > 50%), making sample size relatively small and underpowered. Infarct size was probably decreased by the presence of collaterals, which were present in > 90% of the patients. As with all randomized trials, patient selection has occurred. The mortality rate was relatively low in our study cohort with the risk of overfitting the model and EXPLORE was not powered for clinical outcomes.

## Conclusion

Global and segmental myocardial strains, measured by FT-CMR, improved significantly over time in STEMI patients with a concurrent CTO, with no beneficial effect of CTO-PCI. GLS was an independent predictor for functional recovery, incremental to infarct size, LVEF, and clinical parameters. Although GCS predicted mortality, infarct size remained the strongest predictor. Segmental circumferential strain was an independent predictor for regional LV recovery in the dysfunctional CTO territory.

## Electronic supplementary material

ESM 1 (DOC 1386 kb)

ESM 2 (PPTX 44711 kb)

## Abbreviations

CMR: Cardiovascular magnetic resonance
CTO: Chronic total occlusion
EXPLORE: Evaluating Xience and left ventricular function in PCI on occlusiOns afteR STEMI
FT: Feature tracking
GCS: Global circumferential strain
GLS: Global longitudinal strain
IRA: Infarct-related artery
LGE: Late gadolinium-enhanced
LVEDV: Left ventricular end-diastolic volume
LVEF: Left ventricular ejection fraction
MVO: Microvascular obstruction
PCI: Percutaneous coronary intervention
SSFP: Steady-state free-precision
STEMI: ST-segment elevation myocardial infarction
SWT: Segmental wall thickening
TEI: Transmural extent of infarction
