# Supplementary material for: Recovery and prognostic value of myocardial strain in ST-segment elevation myocardial infarction patients with a concurrent chronic total occlusion
Source: Eur Radiol. 2019 Jul 26;30(1):600–8. doi: 10.1007/s00330-019-06338-x (PMC6890657; doi:10.1007/s00330-019-06338-x)
Supplement: Supplementary file 1 — (DOC 1386 kb) [file 330_2019_6338_MOESM1_ESM.doc]

**Supplemental:**

***Inclusion criteria EXPLORE Trial***

Patients with a non-infarct related chronic total occlusion undergoing successful primary PCI for STEMI (within twelve hours of onset of symptoms) are screened for entry into this trial. For the purpose of this trial, a primary PCI is ‘successful’ when the residual stenosis of the culprit lesion < 30% and the TIMI flow ≥ 2.

STEMI is diagnosed when both of the following apply:

- Typical chest pain
- Electrocardiographic new or presumed new ST segment elevation at the J point in two or more contiguous leads with the cut-off points ≥ 0.2 mV in leads V1, V2, or V3 and ≥ 0.1 mV in other leads.

Patients are suitable for inclusion in this trial if coronary angiography preceding the primary PCI reveals at least one chronic total occlusion with all of the following characteristics:

1. Located in a non-infarct related coronary artery:
   1. In the left coronary system if the right coronary artery (RCA) is the culprit lesion
   2. In the RCA or left circumflex artery (LCX) if the left anterior descending artery (LAD) is culprit lesion;
   3. In the RCA or LAD if the LCX is the culprit lesion.
2. A 100% luminal narrowing without antegrade flow or with antegrade or retrograde filling through collateral vessels
3. Amenable to PCI treatment
4. A reference diameter of ≥ 2.5 millimeters by visual estimation

***Exclusion criteria***

1. Older than 80 years of age
2. Persistent or permanent atrial fibrillation
3. Known renal insufficiency (e.g. serum creatinine level of more than 265 μmol/L (i.e. more than 3.5 mg/L))
4. More than 48 hours of hemodynamic instability after primary PCI, defined as pre-shock (heart rate >100/min. and or systolic blood pressure <100 mmHg) or shock (sustained systolic blood pressure ≤ 80 mmHg despite fluid hydration with ≥ two low dose or one high dose vasopressor or inotropic drug(s) or a cardiac index of ≤ 2.2 liters per minute per square meter of body-surface area and a pulmonary-capillary wedge pressure of at least 15 mmHg if known)
5. Cardiac events between primary PCI and randomization:
   1. Extended myocardial infarction, as evidenced by a new episode of chest pain with new ST-segment elevations and a new CK / CK-MB peak
   2. Acute stent thrombosis
   3. Ventricular arrhythmias, i.e. sustained ventricular tachycardia (VT) or ventricular fibrillation (VF) more than 48 hours after primary PCI (i.e. late ventricular arrhythmia)
6. Significant left main stenosis (diameter stenosis ≥ 50%)
7. Indication for Coronary Artery Bypass Grafting (CABG)
8. Severe valvular heart disease requiring cardiac surgery within four months
9. Indication for implantable cardioverter defibrillator (ICD) within four months
10. Inability to schedule the index procedure within seven days after primary PCI
11. Unsatisfactory baseline investigations, i.e. MRI not suitable for endpoint assessment
12. Any contraindication for MRI, i.e.:
    1. pacemaker
    2. cerebrovascular clips
    3. claustrophobia
13. Serious known concomitant disease with a life expectancy of less than one year
14. Circumstances that prevent follow-up (no permanent home or address, transient, etc.)
15. Previous participation in this trial
16. Current participation in another trial

**Table 1 Supplement: Baseline characteristics of all patients and stratified for randomization outcome.**

|  | | **Total**  **(n=200)** | **CTO-PCI**  **(n=95)** | **No-CTO PCI**  **(n=105)** | **p-value** |
| --- | --- | --- | --- | --- | --- |
| Age (years, mean, SD) |  | 60(10) | 60(10) | 59(10) | 0.61 |
| Male gender |  | 175(88) | 87(92) | 88(84) | 0.13 |
| Diabetes |  | 31(16) | 16(17) ) | 15(14) | 0.70 |
| Hypertension |  | 87(44) | 35(37) | 52(50) | 0.09 |
| Hypercholesterolemia |  | 67(34) | 31(33) | 36(34) | 0.88 |
| Current smoker |  | 107(54) | 51(54) | 56(53) | 1.00 |
| Previous MI |  | 31(16) | 13(14) | 18(17) | 0.56 |
| Baseline LVEF (%, mean, SD) |  | 41(12) | 41(11) | 42(12) | 0.57 |
| Baseline LVEDV (ml/m^2^, mean, SD) |  | 103(25) | 103(24) | 103(25) | 0.92 |
| Baseline infarct size (g)* |  | 12(11) | 12(10) | 12(13) | 0.84 |
| ***Primary PCI*** |  |  |  |  |  |
| **Infarct related artery** |  |  |  |  | 0.09 |
| Right coronary artery |  | 64(32) | 31(33) | 33(31) |  |
| Left circumflex artery |  | 47(24) | 16(17) | 31(30) |  |
| Left anterior descending artery/LM |  | 89(45) | 48(51) | 41(39) ) |  |
| Three vessel disease (>70% stenosis) |  | 87(44) | 41(43) | 46(44) | 1.00 |
| MI SYNTAX score II (mean, SD) (wiring/balloon/aspiration) |  | 27(9) | 27(9) | 26(10) | 0.56 |
| **CTO characteristics** |  |  |  |  |  |
| **CTO related artery** † |  |  |  |  | 0.12 |
| Right coronary artery |  | 93(47) | 37(39) ) | 56(53) |  |
| Left circumflex artery |  | 56(28) | 31(33) | 25(24) |  |
| Left anterior descending artery |  | 51(26) | 27(28) | 24(23) |  |
| Total J-CTO score (mean, SD) |  | 2.1(1.1) | 2.0(1.1) | 2.2(1.2) | 0.15 |

Data are number of patients (%), unless otherwise stated. Baseline characteristics were compared using the independent-samples T-test, or Fisher's exact probability test in case of binary endpoints. *Infarct data was available for 148 patients. † For patients with multiple CTOs, the CTO supplying the largest amount of myocardium was defined as the main CTO. PCI=percutaneous coronary intervention. CTO=chronic total occlusion. LVEF= left ventricular ejection fraction, LVEDV= left ventricular end-diastolic volume. LM=left main artery. MI SYNTAX= Synergy Between Percutaneous Coronary Intervention With Taxus and Cardiac Surgery. MI= myocardial infarction. J-CTO=Multicenter CTO registry of Japan.

**Table 2 Supplement: Global strain and its association with global** left ventricular function

|  | **GLS** | | **GCS** | |
| --- | --- | --- | --- | --- |
|  | **r** | **p-value** | **r** | **p-value** |
| **LVEF (%)** |  |  |  |  |
| **Baseline** | -0.78 | <0.001 | -0.92 | <0.001 |
| **Follow-up** | -0.79 | <0.001 | -0.91 | <0.001 |
| **Change** | -0.45 | <0.001 | -0.70 | <0.001 |
| **LVEDV (ml/m²)** |  |  |  |  |
| **Baseline** | 0.36 | <0.001 | 0.49 | <0.001 |
| **Follow-up** | 0.41 | <0.001 | 0.57 | <0.001 |
| **Change** | -0.006 | 0.94 | 0.14 | 0.09 |
| **Infarct (g)** |  |  |  |  |
| **Baseline** | 0.54 | <0.001 | 0.48 | <0.001 |
| **Follow-up** | 0.46 | <0.001 | 0.56 | <0.001 |
| **Change** | 0.008 | 0.93 | -0.13 | 0.14 |

*Pearson's correlation coefficient analysis was used to assess the relationship between the global strain and LV function parameters (r). GLS= global longitudinal strain. GCS= global circumferential strain. LVEF= left ventricular ejection fraction. LVEDV= left ventricular end-diastolic volume. LVESV= left ventricular end-systolic volume.

**Table 3 Supplement: Prediction of all-cause mortality (n=166)**

|  | **Univariate analysis** | | | **Multivariate analysis** | | |
| --- | --- | --- | --- | --- | --- | --- |
|  | **Hazard ratio** | **95% CI** | **p-value** | **Hazard ratio** | **95% CI** | **p-value** |
| **Age (years)** | 1.05 | 0.98-1.13 | 0.14 | - | - | - |
| **Male** | 1.11 | 0.14-8.90 | 0.92 | - | - | - |
| **Diabetes** | 1.70 | 0.35-8.22 | 0.51 | - | - | - |
| **Infarct LAD** | 1.40 | 0.38-5.22 | 0.62 | - | - | - |
| **CTO LAD** | 2.42 | 0.65-9.02 | 0.19 | - | - | - |
| **CTO-PCI** | 1.30 | 0.35-4.82 | 0.70 | - | - | - |
| **Baseline LVEF (%)** | 0.93 | 0.88-0.98 | 0.01 | - | - | - |
| **Baseline LVEDV (ml/m²)** | 1.02 | 1.00-1.05 | 0.045 | - | - | - |
| **Baseline GLS** | 1.10 | 0.98-1.23 | 0.11 | - | - | - |
| **Baseline GCS** | 1.17 | 1.04-1.31 | 0.009 | 1.17 | 1.04-1.31 | 0.009 |

*Hazard ratios for long-term mortality were calculated using Cox proportional hazard regression analyses. Stepwise forward selection of variables was used for multivariable analysis. CTO=chronic total occlusion, LAD= left anterior descending artery, LVEDV= left ventricular end-diastolic volume, LVEF= left ventricular ejection fraction, PCI= percutaneous coronary intervention. GLS= global longitudinal strain, GCS= global circumferential strain.

**Supplemental figure: Feature-tracking Cardiac Magnetic Resonance Assessment** Global Longitudinal strain and global circumferential strain throughout the cardiac cycle. Per patient, peak global strain (% deformation) was calculated.

**
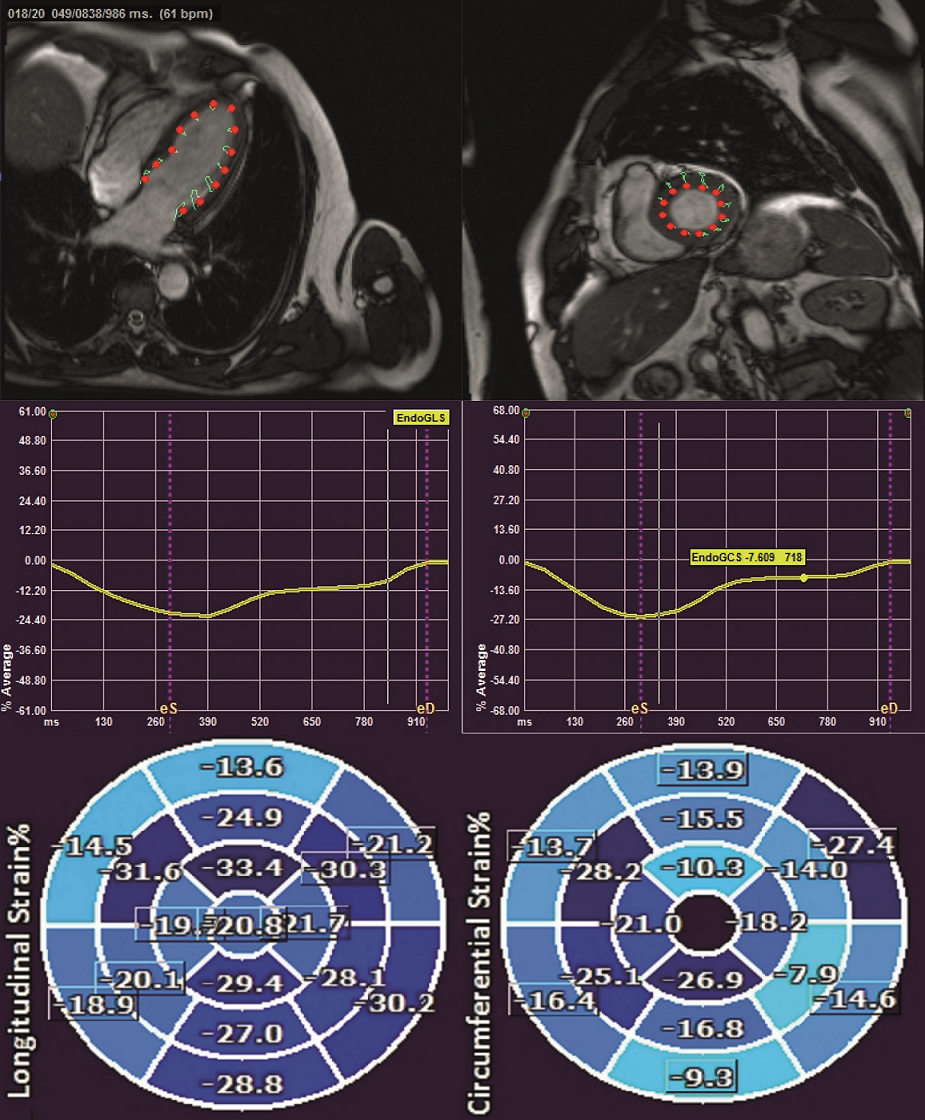
**
