# Supplementary material for: Recovery and prognostic value of myocardial strain in ST-segment elevation myocardial infarction patients with a concurrent chronic total occlusion
Source: Eur Radiol. 2019 Jul 26;30(1):600–8. doi: 10.1007/s00330-019-06338-x (PMC6890657; doi:10.1007/s00330-019-06338-x)
Supplement: Supplementary file 2 — (PPTX 44711 kb) [file 330_2019_6338_MOESM2_ESM.pptx]

## Slide 1
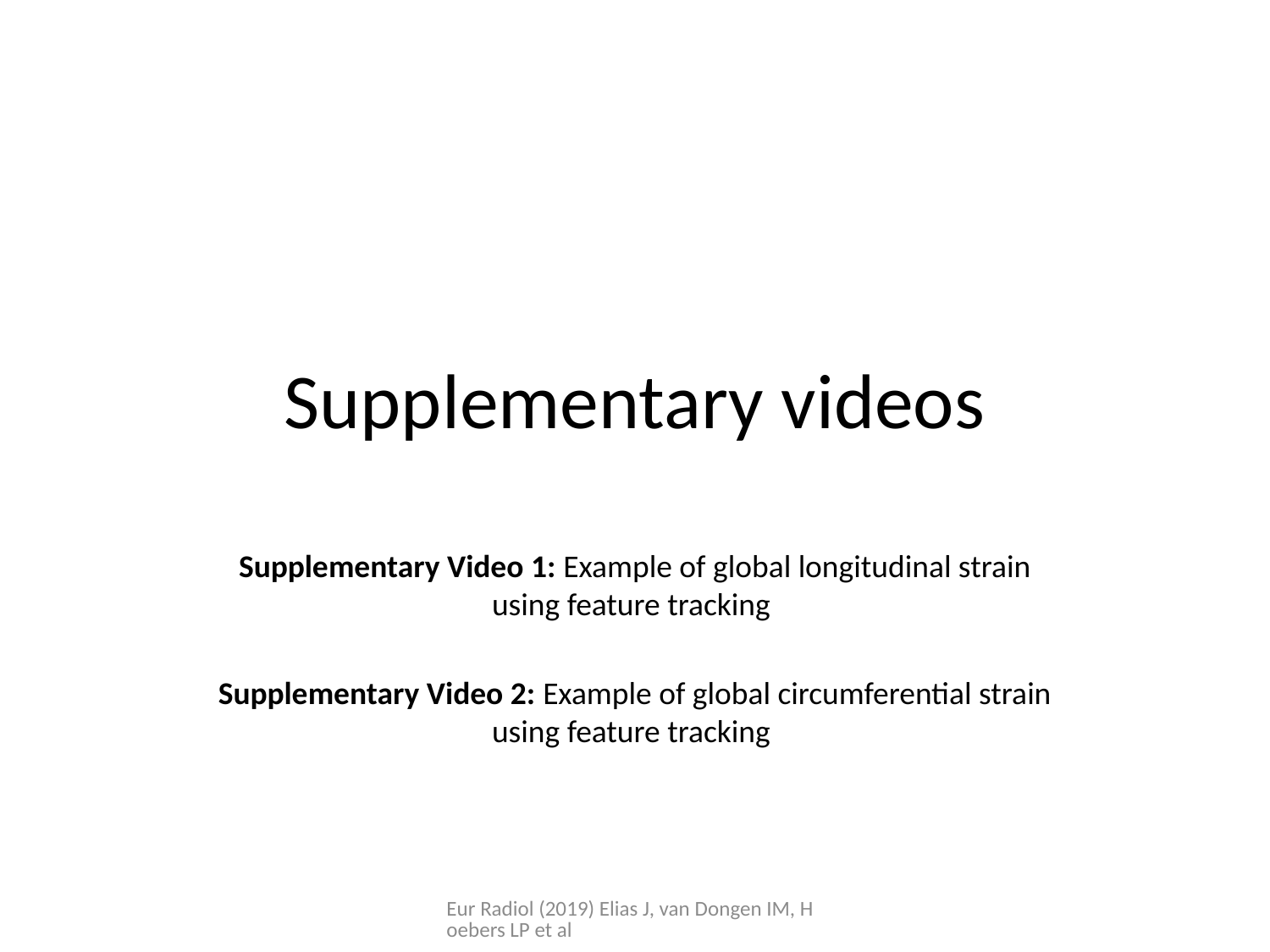

# Supplementary videos
Supplementary Video 1: Example of global longitudinal strain using feature tracking
Supplementary Video 2: Example of global circumferential strain using feature tracking
Eur Radiol (2019) Elias J, van Dongen IM, Hoebers LP et al

## Slide 2
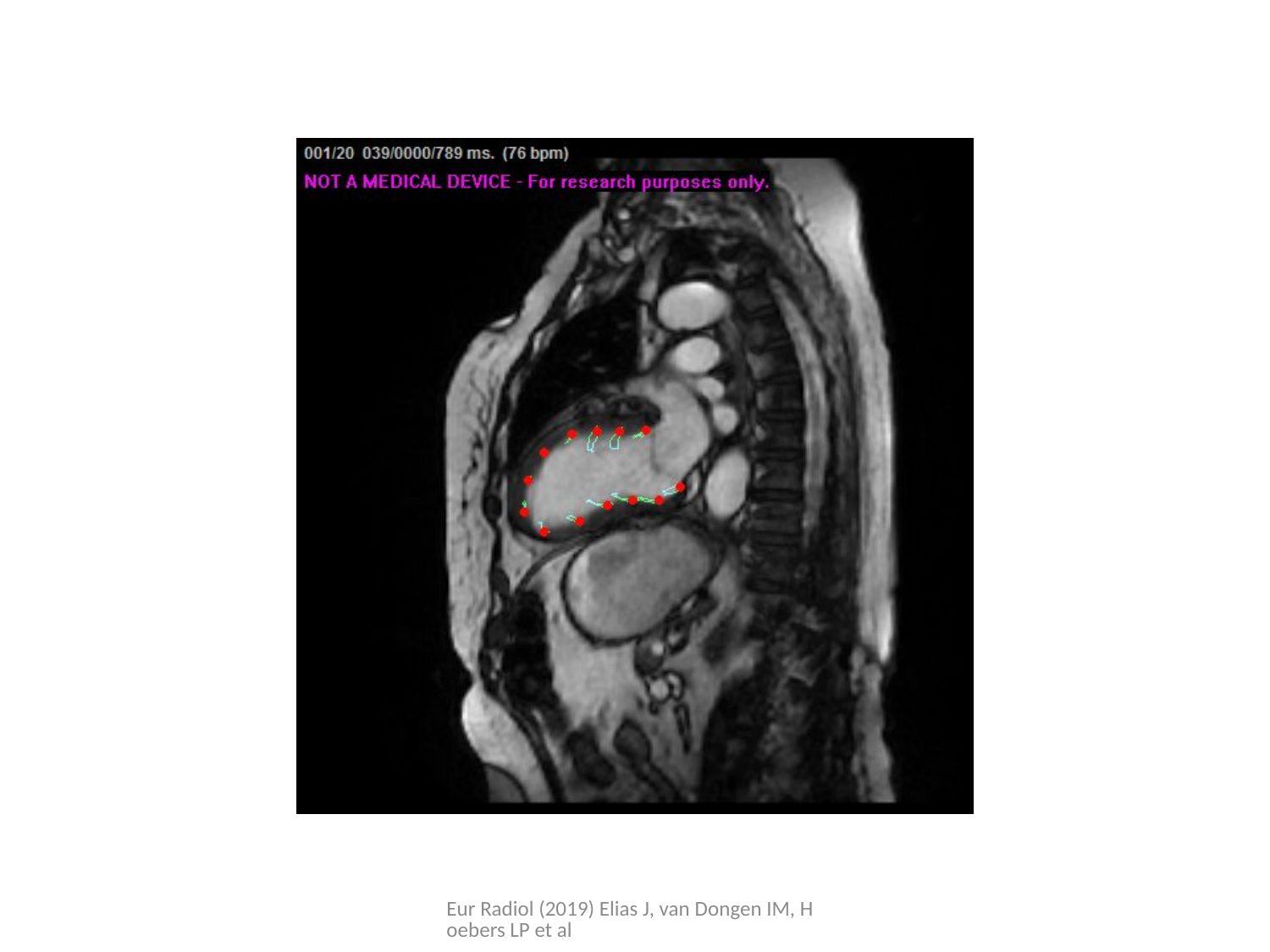

Eur Radiol (2019) Elias J, van Dongen IM, Hoebers LP et al

## Slide 3
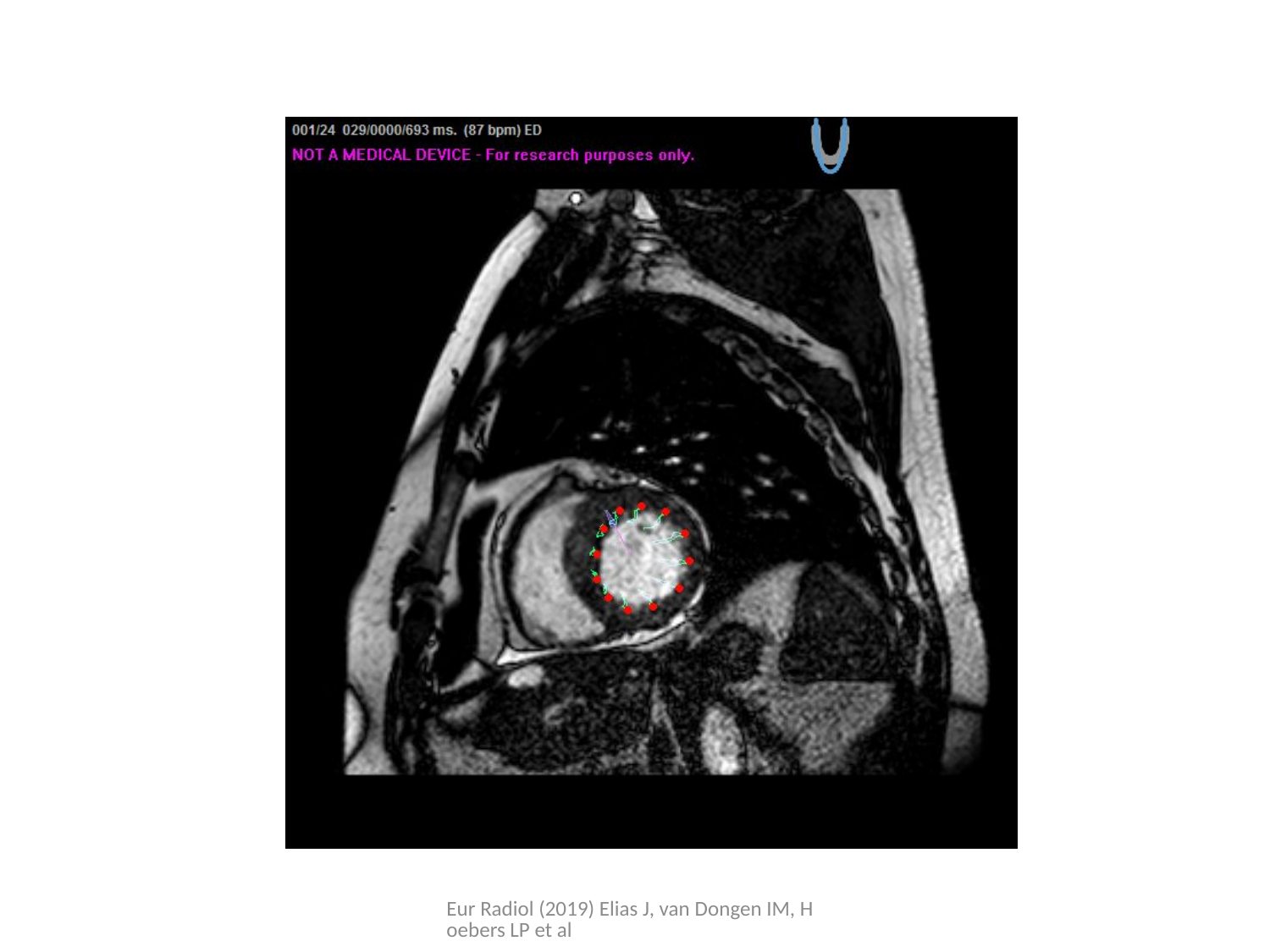

Eur Radiol (2019) Elias J, van Dongen IM, Hoebers LP et al
